# Skin and Colon Cancer Media Campaigns in Utah

**Published:** 2004-09-15

**Authors:** Camille Broadwater, Janet Heins, Catherine Hoelscher, Adam Mangone, Cami Rozanas

**Affiliations:** College of Nursing, University of Utah, Salt Lake City, Utah; Health Program Coordinator, Utah Department of Health; Breast and Cervical Cancer Program, Utah Department of Health, Salt Lake City, Utah; Love Communications, Salt Lake City, Utah; Crowell Advertising, Marketing and PR Agency, Salt Lake City, Utah

## Abstract

The mission of the Utah Cancer Action Network is to reduce cancer incidence and mortality in Utah. Established in 2003, the network selected skin and colon cancers as the first priorities in its comprehensive plan. In its first year of operation, the network planned and implemented a cancer awareness campaign that was organized along two tracks: 1) marketing research, consisting of two telephone surveys, and 2) two advertising/awareness campaigns, one for colon cancer and one for skin cancer. The first telephone survey was conducted in January 2003 to obtain a baseline measurement of the Utah population's knowledge, attitudes, and behaviors. The advertising campaigns were launched in April 2003, and the second telephone survey was conducted in May.

In January 2003, 18% of survey respondents reported seeing or hearing skin cancer prevention or sun protection announcements; in May, this percentage increased to 76%. In January, 36% indicated they had seen, read, or heard colorectal cancer early detection announcements; in May, this percentage increased to 79%.

## Introduction

The Utah Cancer Action Network (UCAN) was established in 2003 with a mission to reduce cancer incidence and mortality in Utah through collaborative efforts that provide services and programs directed toward comprehensive cancer prevention and control. Its open membership includes 72 participating partners ranging from universities to hospitals to the Utah State House of Representatives. Staff from the Utah Department of Health Cancer Control Program provide administrative support to UCAN: two full-time employees and one half-time support employee plan, implement, and monitor program activities.

UCAN is funded by grants from the Centers for Disease Control and Prevention. In fiscal year 2003, UCAN received approximately $1 million: $322,000 for its Comprehensive Cancer core activities, $330,000 for the colon cancer campaign, $330,000 for the skin cancer campaign, and $29,000 for a prostate cancer program.

One of UCAN's primary responsibilities is to recommend priorities for cancer prevention and early detection efforts in Utah. UCAN selected skin and colon cancers as the first priorities in its comprehensive plan. UCAN's goals are consistent with the goals of *Healthy People 2010* (1). One goal is to reduce the incidence of skin cancer in Utah by decreasing the proportion of adults and young people who acquire sunburn to less than 30% by 2005. Another goal is to promote and increase colon cancer screening rates to 50% among people aged 50 or older who have 1) had a fecal occult blood test in the past two years and 2) ever had a sigmoidoscopy.

To that end, in its first year of operation, the network planned and implemented a cancer awareness campaign that was organized along two tracks: 1) marketing research, consisting of two telephone surveys, and 2) two advertising/awareness campaigns (one for colon cancer and one for skin cancer). The goal of the awareness campaign was two-fold: first, to create a brand and messaging strategy to establish the newly formed UCAN as a community cancer prevention leader; and, second, to increase public awareness about the importance of early detection and prevention of colon cancer and skin cancer.

### Skin cancer incidence in Utah

In Utah, people are at increased risk of developing skin cancer because of a predominance of sunny days, a high altitude, and residents with fair skin. Utah has approximately 241 sunny days annually, and the U.S. has an estimated 213 sunny days each year. Utah ranks third among all U.S. states in average elevation (6100 ft). Approximately 89% of the state's population is white, compared with the national average of 75%, for the year 2000 (2). These factors may contribute to higher incidence of skin cancer that consistently exceeds the national average.

According to Surveillance, Epidemiology, and End Results (1996–2000), the age-adjusted incidence rate for melanoma in Utah was 19.93 per 100,000, and the national average was 17.52 per 100,000 (3). Looking forward, it is projected that 420 Utahns will be diagnosed with malignant melanoma in 2004 (4). Additionally, the 2000 Utah Behavioral Risk Factor Surveillance System indicated that nearly 48% of adults reported obtaining sunburn in the previous 12 months (5).

### Colon cancer incidence in Utah

It was estimated that 700 new cases of colon cancer would be diagnosed and 300 Utahns would die from colon cancer Utah in 2003 (6). Colon cancer is most common in men and women aged 50 and older (6,7). The risk increases with age: 93% of cases were diagnosed in people aged 50 years and older (6,7). Colon cancer is the number two cancer killer in both the United States and Utah, and it is more than 90% preventable if properly screened (7).

## The Cancer Awareness Media Campaigns

### Utah Department of Health Cancer Advertising Awareness Survey

To assess public knowledge, attitudes, and health behaviors regarding skin and colon cancers both prior to and after the launch of the UCAN advertising campaigns, UCAN contracted with a local (Salt Lake City) marketing research firm to conduct the 2003 Utah Department of Health Cancer Advertising Awareness Survey. The research firm was selected because of its experience in opinion polls, marketing, customer information, and social and public policy research. Two telephone surveys were administered by the research firm: one in January 2003 and another in May 2003. The sample for both surveys was an equal probability telephone sample of all Utah households. The January survey was designed to obtain a baseline measurement of the Utah population's knowledge, attitudes, and behaviors.

The January survey was developed by the Cancer Program staff in conjunction with the marketing research firm. Prior to administering the survey, a draft survey instrument was prepared and tested among 23 randomly selected Utah residents in a focus group setting. The focus group participants were asked to complete the survey as if they were respondents, and they were also asked to provide feedback on questions, especially if they were unclear about the intent of a question, if there were terms they did not understand, or if the survey format was not clear. Feedback from these pretest interviews helped to formulate the final questionnaire.

January survey data collection began on January 20, 2003, and concluded by January 31. All sampling during the course of the survey research relied on random-digit-dial protocols. When a household was contacted, an adult within the household was identified and asked to participate in the survey, which required an average of 10 minutes for participants to complete. The overall survey response rate for the January 2003 administration was 81%, and 816 individuals completed the survey.

The January survey was divided into two target audiences, one for skin cancer, which targeted an audience of adults aged 18–49 with children, and another for colon cancer, which targeted an audience of adults aged 50 and older; 407 individuals completed the skin cancer surveys and 409 individuals completed the colon cancer surveys.

### UCAN advertising campaigns

In February 2003, UCAN contracted with a local advertising firm to develop two advertising campaigns. Incorporating feedback from the January survey, UCAN developed several test messages for both colon and skin cancer. For skin cancer, UCAN focused on the issues of personal risk assessment and protective clothing. For colon cancer, UCAN focused on the perception that colon cancer primarily affects males and that screening is necessary only when symptoms are present. To help test the messages within each target audience, UCAN conducted focus group research, twice for skin cancer and twice for colon cancer. Each focus group consisted of eight or nine participants. Focus group results determined the creative strategy that shaped the key messages.

#### Skin cancer campaign

Utah Skin Cancer Campaign Materials

**Figure:**
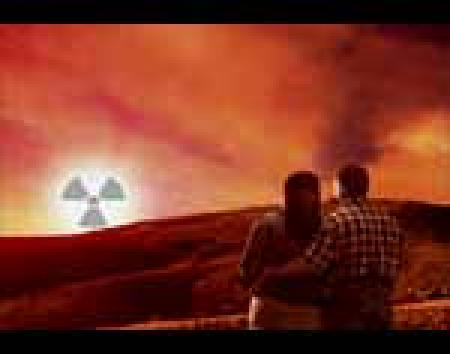
“ADULTS” Watch "Adults" UCAN TV spot (RM 1mb)

10-second TV Spot
GRAPHICS Produced By The CROWELL/LOVE Partnership, Salt Lake City, Utah. Crowell Advertising: 801-531-0533 Love Communications: 801-519-8880 (Couple watching the sunset.)
MALE VOICE Exposure to the sun’s ultraviolet rays accounts for more than half of all cancers in America.
GRAPHICS Don’t take the sun lightly. Cover up or use sunscreen on you and your kids every day. UCAN www.ucan.cc Utah Cancer Action Network (End.)

**Figure:**
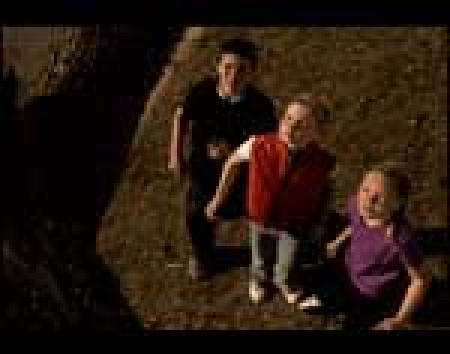
"KIDS" Watch "Kids" UCAN TV spot (RM 2mb)

30-second TV Spot
GRAPHICS Produced By The CROWELL/LOVE Partnership, Salt Lake City, Utah. Crowell Advertising: 801-531-0533 Love Communications: 801-519-8880
GRAPHICS The following commercial shows children being exposed to the world’s largest ultraviolet radiation leak. Due to its graphic nature, parental discretion is advised. (Children running and playing outside.)
MALE VOICE Sun exposure during your child’s first 18 years is the biggest factor in them getting skin cancer. Don’t take the sun lightly. Cover up or use sunscreen on you and your kids every day.
GRAPHICS Don’t take the sun lightly. Cover up or use sunscreen on you and your kids every day. UCAN www.ucan.cc Utah Cancer Action Network (End.)

**Figure:**
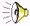
“CLOWN” Radio Spot Hear "Clown" UCAN radio spot (MP3 1.8mb)

(Music.)
NARRATOR Look outside at your children — bicycle helmets, elbow pads, kneepads. Too bad none of these items can protect them from what’s hiding on the roof. Look up. See it? See the clown? Yes, a clown, smiling, with jagged teeth and ill intent at your wee ones, flamethrower in hand. If ultraviolet radiation looked like this, you’d never let your children outside again — ever. Unfortunately, the sun’s ultraviolet radiation doesn’t look like anything at all. It’s invisible. Yet, every day, this unseen killer punches through the earth’s atmosphere, through the clouds, and into your child’s flesh, eventually rearing its ugly head again as skin cancer. If you want to protect your family, don’t take the sun lightly. Cover up or use sunscreen on you and your children every day. Learn more at the Utah Cancer Action Network Web site, Ucan.cc.
(End.)

**Figure:**
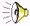
“SPIDER” Radio Spot Hear "Spider" UCAN radio spot (MP3 1.8mb)

(Music.)
NARRATOR Your children have little business playing in this park. After all, this park is home to one of nature’s monsters, the flying black widow spider. Larger than an orange, this deadly spider lurks in treetops and — wait, there’s one now, landing on your child’s bare neck. If ultraviolet radiation looked like this, you’d never let your children outside again — ever. Unfortunately, the sun’s ultraviolet radiation doesn’t look like anything at all. It’s invisible. Yet, every day, this unseen killer attacks your child. And the more ultraviolet radiation your child receives in their first 18 years, the more likely it is they’ll develop skin cancer as an adult. If you want to protect your family, don’t take the sun lightly. Cover up or use sunscreen on you and your children every day. Learn more at the Utah Cancer Action Network Web site, Ucan.cc.
(End.)

Take a tour of the UCAN skin cancer print materials

**Figure:**
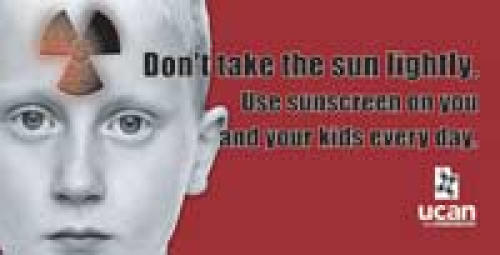
Kids and Sunscreen Billboard VISUAL: A photograph of a child’s solemn face. On his forehead glows the warning symbol for radiation. HEADLINE COPY: Don’t take the sun lightly. Use sunscreen on you and your kids every day. VISUAL: UCAN LOGO UCAN Utah Cancer Action Network

**Figure:**
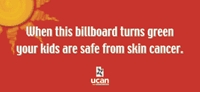
Turning Green Billboard VISUAL: A cartoon sun on a red billboard. HEADLINE COPY: When this billboard turns green your kids are safe from skin cancer. VISUAL: UCAN LOGO UCAN Utah Cancer Action Network

**Figure:**
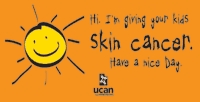
Skin Cancer Billboard VISUAL: A cheery cartoon sun with a smiling face. HEADLINE COPY: Hi. I’m giving your kids skin cancer. Have a nice day. VISUAL: UCAN LOGO Utah Cancer Action Network

**Figure:**
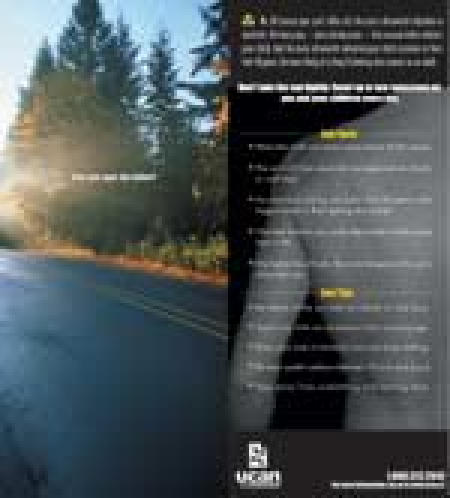
Spot the Killer Rack Card LEFT SIDE OF CARD VISUAL: Photograph of an empty country road bathed in warm sunlight. HEADLINE COPY: Can you spot the killer? RIGHT SIDE OF CARD VISUAL: Photograph of a person’s freckled back. COPY: No. Of course you can’t. After all, the sun’s ultraviolet radiation is invisible. Yet every day — even cloudy ones — this unseen killer attacks your child. And the more ultraviolet radiation your child receives in their first 18 years, the more likely it is they’ll develop skin cancer as an adult. Sun Facts: More time in the sun means more chance of skin cancer.The sun’s UV rays cause skin damage even on cloudy or cold days.Sun exposure during your kids’ first 18 years is the biggest factor in them getting skin cancer.One bad sunburn as a child often results in skin cancer later in life.The higher the altitude, the more dangerous the sun’s ultraviolet rays. Sun Tips: Be aware of the sun even on cloudy or cold days.Teach your kids sun protection from a young age.Dress your kids in brimmed hats and long clothing.Be most careful outdoors between 10 a.m. and 4 p.m.Stay away from sunbathing and tanning beds. VISUAL: UCAN LOGO UCAN Utah Cancer Action Network 1-888-222-2542 For more information, log on to www.ucan.cc

Utah Colon Cancer Campaign Materials

**Figure:**
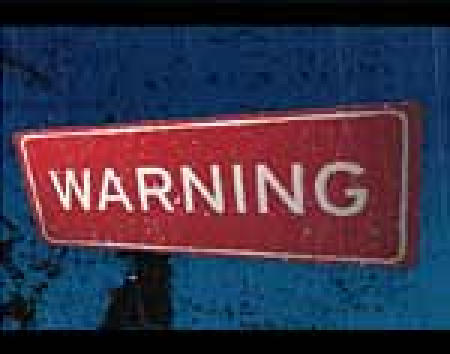
"RUNNER" Watch "Runner" UCAN TV spot (RM 2mb)

30-second TV Spot
GRAPHICS Produced By The CROWELL/LOVE Partnership, Salt Lake City, Utah. Crowell Advertising: 801-531-0533 Love Communications: 801-519-8880 (Couple watching the sunset.)
GRAPHICS WARNING SIGNS OF COLON CANCER VIEWER DISCRETION ADVISED
MALE VOICE Warning, you’re about to see the early warning signs of colon cancer. Viewer discretion is advised. (Music.)

(Couple dancing.)

(Couple dining.)

(Male jogging.)
GRAPHICS FACT: NO WARNING SIGNS
MALE VOICE The fact is, often there are no early warning signs of colon cancer. But the number two cancer killer in Utah is preventable. So, if you’re 50 or older, call your doctor to find out which colon cancer screening option is right for you. Or call this number for more information.
GRAPHICS UCAN Utah Cancer Action Network 1-888-222-2542 Health Resource Line (End.)

**Figure:**
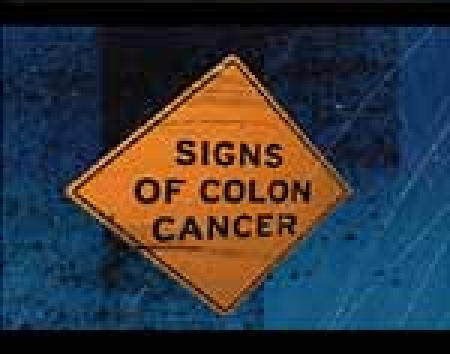
"WALKING" Watch "Walking" UCAN TV spot (RM 1.1mb)

15-second TV Spot
GRAPHICS Produced By The CROWELL/LOVE Partnership, Salt Lake City, Utah. Crowell Advertising: 801-531-0533 Love Communications: 801-519-8880
GRAPHICS WARNING SIGNS OF COLON CANCER VIEWER DISCRETION ADVISED
MALE VOICE Warning, you’re about to see the early warning signs of colon cancer. Viewer discretion is advised. (Music.)

(Couple walking.)
GRAPHICS FACT: NO WARNING SIGNS
MALE VOICE The fact is, often there are no early warning signs. If you’re 50 or older, call your doctor today for a colon cancer screening, or call this number.
GRAPHICS UCAN Utah Cancer Action Network 1-888-222-2542 Health Resource Line (End.)

**Figure:**
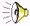
“Dr. Bob” Radio Spot Hear "Dr. Bob" UCAN radio spot (MP3 1.1mb)

(Music.)
DR. BOB Hello, I’m Dr. Bob. And welcome to Health Beat Radio. Today we’re talking about the early warning signs of colon cancer. (Rustling paper.)
DR. BOB Okay, what’s going on here? Where’s my script?
MALE VOICE Well, Dr. Bob, without a colon cancer screening test, there are no early warning signs.
DR. BOB Oh, come on, there have to be early warning signs, like — like what about cramping?
MALE VOICE Nope.
DR. BOB Or bloody, well, you know — derriere (whispers the word)?
MALE VOICE Uhn-uhn.
DR. BOB Fever?
MALE VOICE We’re not talking flu, Dr. Bob.
DR. BOB You got a point. But there has to be pain or something somewhere, doesn’t there?
MALE VOICE By the time there are symptoms, it might be too late.
DR. BOB Ahh.
MALE VOICE Now, exactly what are you a doctor of, Dr. Bob?
FEMALE VOICE Colon cancer, the number two cancer killer in Utah, is preventable if you get regular colon cancer screening tests. So, if you’re 50 or older, call your physician to find out which screening option is right for you. Or call the UCAN health resource line at 1-888-222-2542. Brought to you by UCAN, Utah Cancer Action Network.
(End.)

**Figure:**
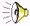
“Warnings” Radio Spot Hear "Warnings" UCAN radio spot (MP3 1.1mb)

(Whistling.)

(Bang.)
MALE VOICE Hey, look out for the pole there.
MALE VOICE Ow!
FEMALE VOICE Sometimes warnings come too late. (Sound of golf ball being struck.)

(Sound of golf ball striking someone.)
MALE VOICE Ow!
MALE VOICE Fore!
FEMALE VOICE And by the time you have colon cancer warning signs you can detect for yourself, it just might be too late. (Sound of chair cracking.)
FEMALE VOICE Whoa! Oh!
FEMALE VOICE Be careful, that chair is broken.
FEMALE VOICE Uhh.
FEMALE VOICE But fortunately, regularly scheduled screening tests prevent colon cancer, the number two cancer killer in Utah. So, if you’re 50 or older, call your doctor to find out which screening option is right for you. Or call the UCAN health resource line at 1-888-222-2542. (Sound of an automobile.)
MALE VOICE Hey, look out for that car! (Sound of automobile horn.)
FEMALE VOICE Whoa! Whew! That was close. Thanks.
FEMALE VOICE Brought to you by UCAN, Utah Cancer Action Network.
(End.)

Take a tour of the UCAN colon cancer print materials

**Figure:**
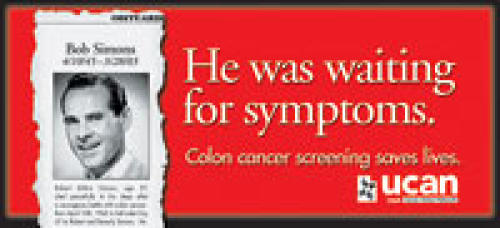
Bob Simons Obit Billboard VISUAL: The top part of an obituary, torn from a newspaper, shows a photograph of a nice-looking man and the first paragraph of the obituary text. COPY FOR VISUAL: Obituary[ies] Bob Simons 4/10/45–3/28/03 Robert Milton Simons, age 57, died peacefully in his sleep after a courageous battle with colon cancer. Born April 10th, 1945, in Salt Lake City, UT, to Robert and Beverly Simons. He... HEADLINE COPY: He was waiting for symptoms. COPY: Colon cancer screening saves lives. VISUAL: UCAN LOGO UCAN Utah Cancer Action Network

**Figure:**
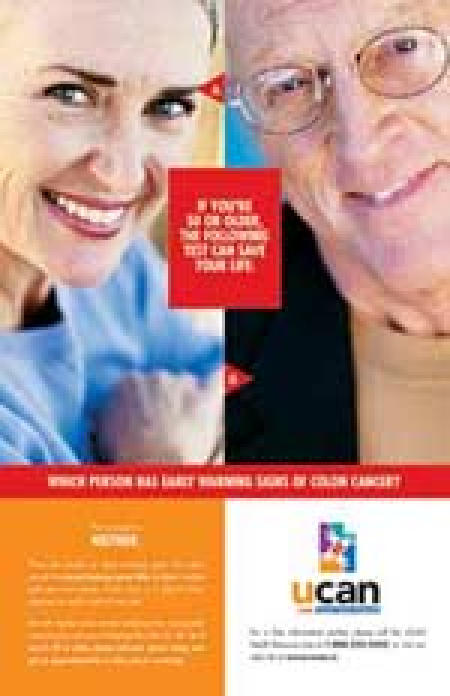
Early Warning Poster VISUAL: Pleasant, smiling faces of two late-middle-aged people, one female (labeled A with an arrow) and one male (labeled B). HEADLINE COPY: When this billboard turns green your kids are safe from skin cancer. COPY: The answer is: neither. There are usually no early warning signs with colon cancer, the second leading cancer killer in Utah. It strikes both men and women of all races, so it doesn’t draw attention to itself — until it’s too late. But with regular colon cancer screening tests, you greatly improve your chances of beating this killer for life. So if you’re 50 or older, please call your doctor today and get an appointment for a colon cancer screening. VISUAL: UCAN LOGO UCAN Utah Cancer Action Network COPY: For a free information packet, please call the UCAN Health Resource Line at 1-888-222-2542, or visit our web site at www.ucan.cc.

**Figure:**
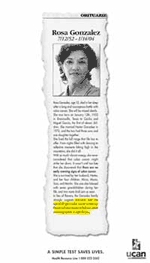
Rosa Gonzalez Obituary Ad VISUAL: Entire obituary, including a photograph of a pleasant-looking middle-aged woman, is shown torn from a newspaper. Copy: Obituary[ies] Rosa Gonzalez 7/12/52–1/14/04 Rosa Gonzalez, age 52, died in her sleep after a long and courageous battle with colon cancer. She will be missed dearly. She was born on January 12th, 1952, in Brownsville, Texas, to Cecilia and Miguel Garcia, the first of eleven children. She married Hector Gonzalez in 1972, and the two had three sons and one daughter together. She lived the full range that life has to offer. From nights filled with dancing to reflective moments hiking high in the mountains, she did it all. With so much vibrant energy, she never considered that colon cancer might strike her down. It wasn’t until too late that she discovered that there are no warning signs of colon cancer. She is survived by her husband, Hector, and her four children: Alicia, Maria, Tara, and Martin. She was also blessed with seven grandchildren during her life, and two more shall join us soon. In lieu of flowers, the Gonzalez family strongly suggests everyone over the age of 50 get a colon cancer screening. Please call your doctor to find out which screening option is right for you. VISUAL: UCAN LOGO Utah Cancer Action Network A SIMPLE TEST SAVES LIVES. Health Resource Line 1-888-222-2542

**Figure:**
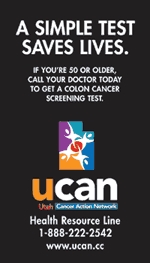
A Simple Test Magnet VISUAL: UCAN (Utah Cancer Action Network) logo HEADLINE COPY: A SIMPLE TEST SAVES LIVES. COPY: If you’re 50 or older, call your doctor today to get a colon cancer screening test. Visual: UCAN LOGO UCAN Utah Cancer Action Network Health Resource Line 1-888-222-2542 www.ucan.cc

The primary messages developed by the advertising firm for UCAN's skin cancer campaign are "Don't take the sun lightly" and "Cover up or use sunscreen on yourself and your children every day." The campaign's take-home message is that parents would protect their children if they realized the dangers of sun exposure. The campaign was launched on April 1, 2003, and the accompanying integrated campaign materials included radio and television ads, print ads, banners, billboards, and collateral materials such as posters, rack cards, water bottles, lip balm, and sunscreen packets.

To maximize the campaign's budget and exposure, UCAN solicited donated space and airtime from selected media outlets. For every dollar spent, two dollars were donated in airtime or promotional value. These donations included Internet exposure, radio remotes, tie-ins with entertainment and sporting events, and weather forecast sponsorships. In addition, for each hour of paid staff time, the advertising firm donated one hour of staff time. We also forged contacts with businesses and community organizations — including parent-teacher associations, WIC (Special Supplemental Nutrition Program for Women, Infants, and Children), Utah State Parks, pharmacies, physicians, local health departments, utility companies, fast food chains, and retail outlets — to disseminate materials and circulate the message within the community.

#### Colon cancer campaign

The colon cancer campaign was launched on April 1, 2003. The key messages developed by the advertising agency were:

- "The fact is, there are no early warning signs of colon cancer."
- "If you're 50 or older, call your doctor to find out which colon cancer screening option is right for you."
- "A simple test saves lives."

Those messages were implemented as part of an integrated and comprehensive marketing strategy. Television, radio, print, public relations, and grassroots efforts targeted an audience aged 45 and older. Local media talent, which appealed to our target demographic group, were recruited as spokespeople and were used to help break down the social stigma surrounding colon cancer. Physicians made public appearances and provided interviews to validate both UCAN and its message. Additionally, Utah's local ABC Television affiliate broadcast a live colonoscopy to eradicate myths about the procedure and show that it is simple and painless.

A major success for the colon campaign was the ability of UCAN to negotiate partnerships that resulted in a three-to-one match for every media dollar spent. Radio and television stations across the state freely promoted UCAN's colon cancer message through local programming, event sponsorship, and news stories. The two largest newspapers in the state printed at no cost a twelve-page UCAN tabloid insert that contained articles written by doctors and health experts from around the state.

Finally, the colon cancer campaign was supported by a grassroots effort that penetrated the community through parent-teacher associations, businesses, physicians, local health departments, and event sponsorships that included the Huntsman Senior World Games in St. George, Utah.

### May follow-up survey

In May, four to six weeks after both campaigns had been launched, another pair of surveys was administered to assess public knowledge, attitudes, and health behaviors regarding skin and colon cancers; 426 adults aged 18 to 49 completed the post-campaign skin cancer surveys and 403 adults aged 50 and older completed post-campaign colon cancer surveys. The May follow-up survey began on May 23, 2003, and concluded on June 9. The response rate for the May 2003 administration was 68%.

### Key research findings

Key research findings are summarized in Table 1 and Table 2. In January 2003, 18% of survey respondents reported seeing or hearing skin cancer prevention or sun protection announcements. The May follow-up survey showed that in less than four weeks on air, recall of UCAN skin cancer ads reached 76%. Among those recalling ads, 78% could "play back" the main message or other specific ad content.

In January 2003, only 36% of survey respondents had heard or seen advertising about colon cancer. This percentage more than doubled to 79% in May. Of the 79% that had heard or seen an ad, 85% could recall of one of UCAN's main messages.

In addition to increasing awareness of UCAN's message through advertising, we also generated favorable publicity. The skin cancer public-relations effort generated more than 33 newspaper articles statewide, 14 television interviews and appearances, and more than a dozen radio interviews with physicians, cancer survivors, and health care professionals.

## Conclusions

Awareness campaigns for skin cancer prevention and early colon cancer detection clarify important issues for the public and help move them toward appropriate health behaviors. The initial print, radio, and television campaign continues to expand and reach additional Utahns. Although this is the initial data reported for the Utah statewide media campaign, time and funds have been allocated to do annual follow-up surveys.

Utah received funds to continue the two media campaigns into a second year. For the second year, the funding level for colon cancer remained the same, but only one-sixth of the original funds was available for skin cancer. A grant application for funds for a third year has been submitted and is pending approval. The telephone post-campaign survey data for year two are being collected. Also, campaign pieces have been offered for use in other communities throughout the United States.

## Figures and Tables

**Table 1:** Characteristics of Four Communities Studied as Part of Regional Health Care System Partnership, North Carolina, 2001

**January 2003 (n = 409)**	**May 2003 (n = 403)**
36% indicated they had seen, read, or heard colorectal cancer^a^ early detection announcements during the past three months.	79% indicated they had seen, read, or heard colon cancer^a^ early detection commercials or ads during the past three months.
Of those who reported seeing, hearing, or reading colorectal announcements, 54% saw an announcement on television, 7% heard it on the radio, and 33% saw it in a newspaper or magazine.	Of those who reported seeing, hearing, or reading colorectal announcements, nearly 86% reported seeing an announcement on television, 15% heard it on the radio, and 18% saw it in a newspaper or magazine.
29% of those seeing or hearing advertising on television or radio did so between 6 PM and 10 PM.	45% of those seeing or hearing advertising on television or radio did so between 6 PM and 10 PM.
Among those recalling advertising, 38% recalled that the message was about getting checkups, getting frequent checkups, or getting tested.	Among those recalling advertising, nearly 31% recalled the message “If 50 or older, call your doctor to find out which colon cancer screening option is right for you.” 25% recalled the message “Fact is, often there are no early warning signs of colon cancer.” 21% recalled the message “Warning — You are about to see the early warning signs of colon cancer.”
Among those recalling advertising, 13% indicated they changed their behavior because of the information in the announcement.	Among those recalling advertising, 7% indicated they changed their behavior because of the information in the announcement.
Among those who did change their behavior, 37% of respondents scheduled a screening, and 5% got additional information about cancer.	Among those who did change their behavior, 35% scheduled an exam and 14% got additional information about colon cancer.
Among those who did not change their behavior, 65% indicated they already did what was recommended.	Among those who did not change their behavior, 51% indicated they already did what was recommended.

a The word “colorectal” in the January survey was changed to “colon” for the May survey.

**Table 2:** Intervention Matrix for Four Communities Participating in Regional Health Care System Partnership, North Carolina, 2001

**January 2003 (n = 407) **	**May 2003 (n = 426) **
18% of respondents reported seeing, hearing, or reading skin cancer prevention or sun protection announcements in the past three months.	76% reported they had seen, read, or heard a skin cancer prevention ad or commercial in the past three months.
Of those who reported seeing, hearing, or reading a skin cancer announcement, 50% reported seeing one on television, 6% heard it on the radio, and 51% saw it in a newspaper or magazine.	Of those who reported seeing, hearing, or reading skin cancer messages, 86% reported seeing one on television, 17% heard it on the radio, and 9% saw it in a newspaper or magazine.
Among those seeing, hearing, or reading an announcement, 40% did so between 6 PM and 10 PM.	Among those seeing, hearing, or reading an announcement, 42% did so between 6 PM and 10 PM.
Among those recalling announcement, 55% indicated the message was to wear sunscreen.	Among those recalling advertising, 24% recalled the message “Don't take the sun lightly. Cover-up or use sunscreen on you and your children every day.” 15% recalled the message “People being penetrated by ultraviolet radiation.” 15% recalled “Children being exposed to world's largest radiation leak.”
32% of survey respondents indicated they changed their behavior based on information in the announcement.	28% of survey respondents indicated they changed their behavior based on information in the announcement.
Among those changing their behavior, 61% indicated they started using sunscreen, 13% started to wear more protective clothing, and 30% became more careful about sun protection for children.	Among those changing their behavior, 60% started using sunscreen or using it more frequently, 23% started to wear more protective clothing, and 19% became more careful about sun protection for children
Among those who did not change behavior, 61% indicated they already did what was recommended.	Among those who did not change their behavior, 49% indicated they already did what was recommended.
