# Supplementary material for: Skin and Colon Cancer Media Campaigns in Utah
Source: Prev Chronic Dis. 2004 Sep 15;1(4):A18. (PMC1277958)
Supplement: Supplementary file 5 [file 04_0023_09.pdf]

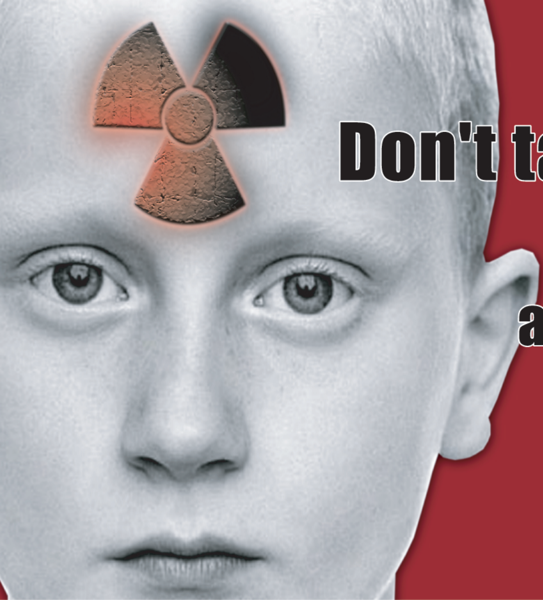

Don't take the sun lightly.

Use sunscreen on you and your kids every day.

ucan

Utah Cancer Action Network
