# Supplementary figures and images for: Skin and Colon Cancer Media Campaigns in Utah
Source: Prev Chronic Dis. 2004 Sep 15;1(4):A18. (PMC1277958)

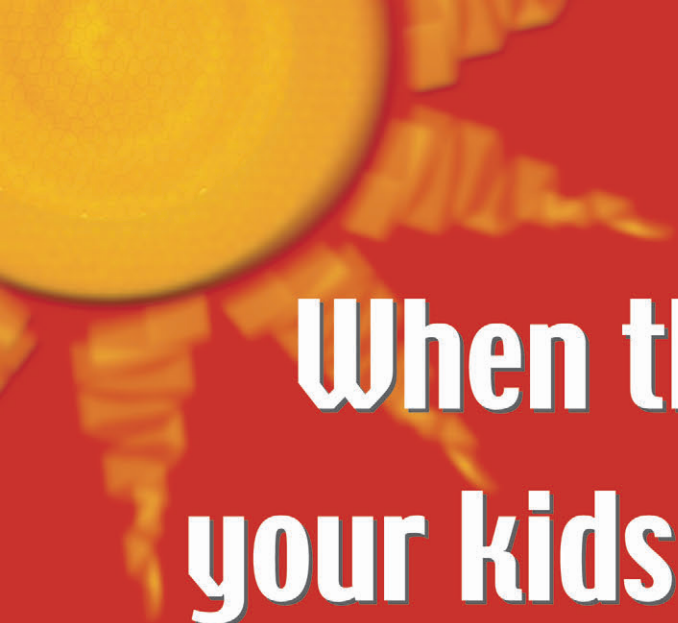

**When this billboard turns green  
your kids are safe from skin cancer.**

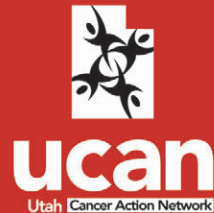

Supplement: Supplementary file 6 [file 04_0023_10.pdf]

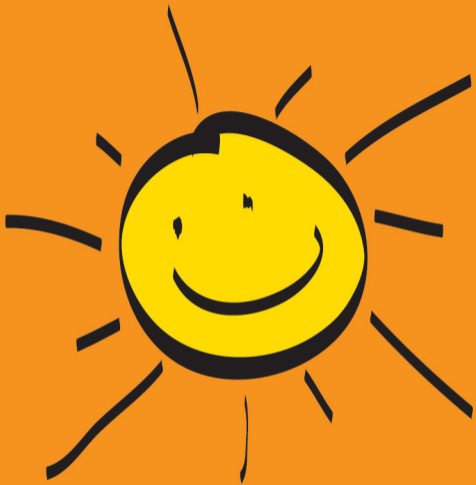

Hi. I'm giving your kids  
**Skin cancer.**  
Have a nice Day.

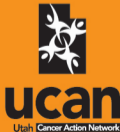

Supplement: Supplementary file 7 [file 04_0023_11.pdf]
