# Supplementary material for: Skin and Colon Cancer Media Campaigns in Utah
Source: Prev Chronic Dis. 2004 Sep 15;1(4):A18. (PMC1277958)
Supplement: Supplementary file 8 [file 04_0023_12.pdf]

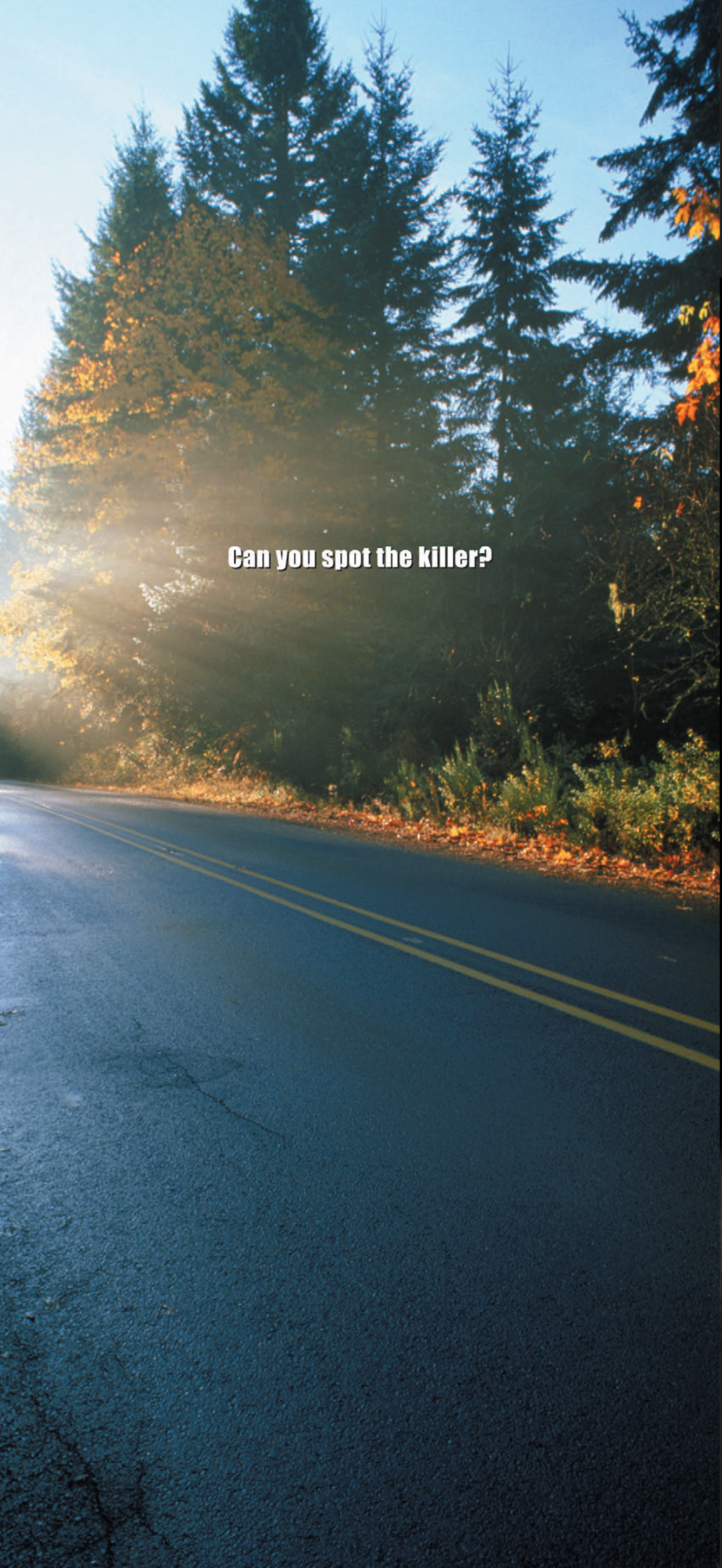

No. Of course you can't. After all, the sun's ultraviolet radiation is invisible. Yet every day -- even cloudy ones -- this unseen killer attacks your child. And the more ultraviolet radiation your child receives in their first 18 years, the more likely it is they'll develop skin cancer as an adult.

Don't take the sun lightly. Cover up or use sunscreen on you and your children every day.

Sun Facts

- More time in the sun means more chance of skin cancer.
- The sun's UV rays cause skin damage even on cloudy or cold days.
- Sun exposure during your kids' first 18 years is the biggest factor in them getting skin cancer.
- One bad sunburn as a child often results in skin cancer later in life.
- The higher the altitude, the more dangerous the sun's ultraviolet rays.

Sun Tips

- Be aware of the sun even on cloudy or cold days.
- Teach your kids sun protection from a young age.
- Dress your kids in brimmed hats and long clothing.
- Be most careful outdoors between 10 a.m. and 4 p.m.
- Stay away from sunbathing and tanning beds.

ucan
Utah Cancer Action Network

1.888.222.2542
For more information, log on to www.ucan.cc
