# Supplementary material for: Skin and Colon Cancer Media Campaigns in Utah
Source: Prev Chronic Dis. 2004 Sep 15;1(4):A18. (PMC1277958)
Supplement: Supplementary file 12 [file 04_0023_01.pdf]

OBITUARIE

## Bob Simons

*4/10/45 - 3/28/03*

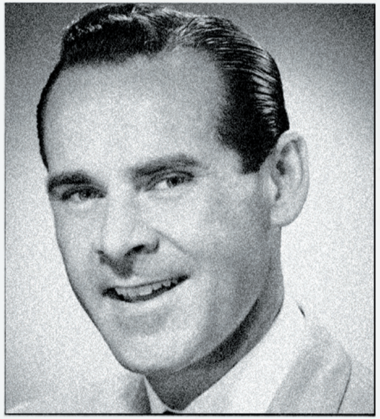

Robert Milton Simons, age 57, died peacefully in his sleep after a courageous battle with colon cancer. Born April 10th, 1945 in Salt Lake City, UT to Robert and Beverly Simons. He

# He was waiting for symptoms.

Colon cancer screening saves lives.

ucan
Utah Cancer Action Network
