# Supplementary material for: Skin and Colon Cancer Media Campaigns in Utah
Source: Prev Chronic Dis. 2004 Sep 15;1(4):A18. (PMC1277958)
Supplement: Supplementary file 13 [file 04_0023_04.pdf]

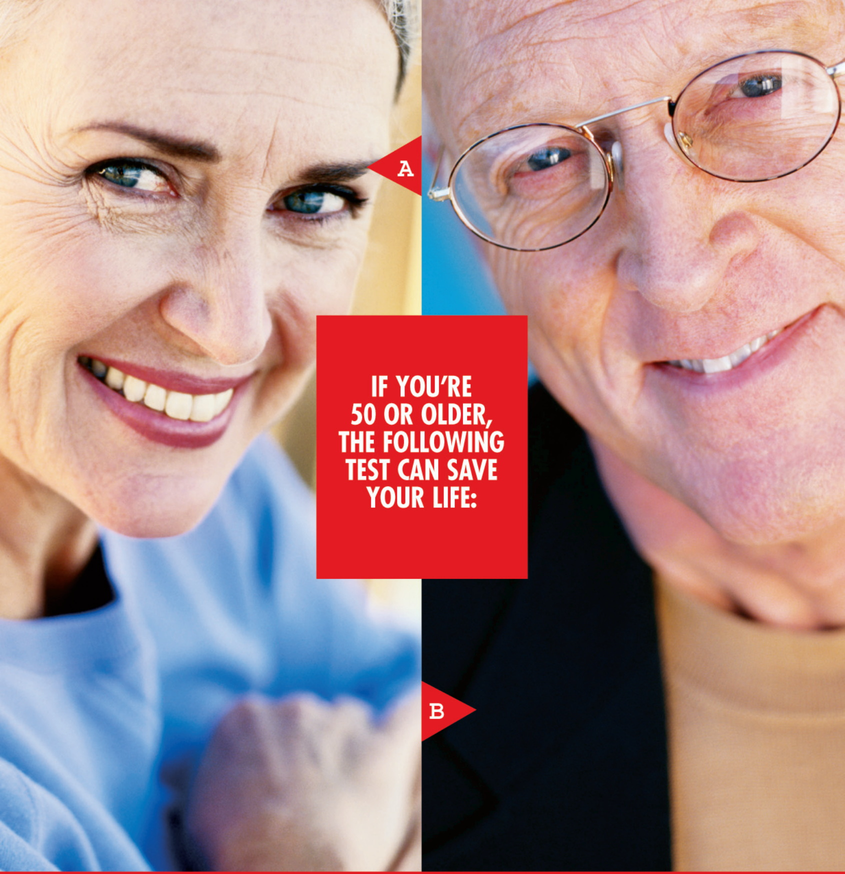

WHICH PERSON HAS EARLY WARNING SIGNS OF COLON CANCER?

The answer is:
NEITHER.

There are usually no early warning signs with colon cancer, the **second leading cancer killer in Utah**. It strikes both men and women of all races, so it doesn't draw attention to itself—until it's too late.

But with regular colon cancer screening tests, you greatly improve your chances of beating this killer for life. **So if you're 50 or older, please call your doctor today and get an appointment for a colon cancer screening.**

ucan
Utah Cancer Action Network

For a free information packet, please call the UCAN Health Resource Line at **1-888-222-2542**, or visit our web site at **www.ucan.cc**.
