# Supplementary material for: Skin and Colon Cancer Media Campaigns in Utah
Source: Prev Chronic Dis. 2004 Sep 15;1(4):A18. (PMC1277958)
Supplement: Supplementary file 14 [file 04_0023_03.pdf]

OBITUARIE

Rosa Gonzalez

7/12/52 ~ 1/14/04

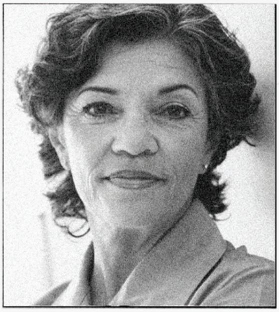

Rosa Gonzalez, age 52, died in her sleep after a long and courageous battle with colon cancer. She will be missed dearly.

She was born on January 12th, 1952 in Brownsville, Texas to Cecilia and Miguel Garcia, the first of eleven children. She married Hector Gonzalez in 1972, and the two had three sons and one daughter together.

She lived the full range that life has to offer. From nights filled with dancing to reflective moments hiking high in the mountains, she did it all.

With so much vibrant energy, she never considered that colon cancer might strike her down. It wasn't until too late that she discovered that **there are no early warning signs of colon cancer.**

She is survived by her husband, Hector, and her four children: Alicia, Maria, Tara, and Martin. She was also blessed with seven grandchildren during her life, and two more shall join us soon.

In lieu of flowers, the Gonzalez family strongly suggests everyone over the age of 50 get a colon cancer screening. Please call your doctor to find out which screening option is right for you.

A SIMPLE TEST SAVES LIVES.

Health Resource Line 1-888-222-2542

ucan

Utah Cancer Action Network
