# Supplementary material for: Skin and Colon Cancer Media Campaigns in Utah
Source: Prev Chronic Dis. 2004 Sep 15;1(4):A18. (PMC1277958)
Supplement: Supplementary file 15 [file 04_0023_02.pdf]

A SIMPLE TEST SAVES LIVES.

IF YOU'RE 50 OR OLDER, CALL YOUR DOCTOR TODAY TO GET A COLON CANCER SCREENING TEST.

ucan

Utah Cancer Action Network

Health Resource Line
1-888-222-2542

www.ucan.cc
